# Dietary Antioxidants and Brain Health: Focus on Cognitive and Affective Disorders

**DOI:** 10.3390/antiox10111659

**Published:** 2021-10-22

**Authors:** Justyna Godos, Giuseppe Grosso

**Affiliations:** Department of Biomedical and Biotechnological Sciences, University of Catania, 95123 Catania, Italy; justyna.godos@gmail.com

Today’s society faces major global challenges, including the continuously increasing prevalence of mental disorders and neurodegenerative diseases, with different risk factors contributing to the trend [1]. As a result, targeting modifiable lifestyle habits, including diet, has been recognized as an essential strategy to reduce this burden [2].

Dietary antioxidants, such as polyphenols, which are bioactive compounds characteristically contained in plant-based diets, have been demonstrated to play a role in the prevention of numerous non-communicable diseases, including cardiovascular diseases [3], hypertension [4], diabetes [5] and cancer [6,7]. Interestingly, recent evidence has suggested that dietary polyphenols may also be implicated in brain health through both indirect and direct mechanisms, including, but not limited to, modulation of neuro-and systemic inflammation, adult neurogenesis, cerebrovascular function, as well as gut microbiota [8,9].

This Special Issue focused on providing literature syntheses and new insights into the effects of antioxidant molecules, as well as antioxidant-rich foods and dietary patterns, toward neurodegenerative diseases and mental disorders.

Among the published articles, two intervention studies explored chronic and acute effects of polyphenol-containing extracts on cognitive function [10,11]. One cross-sectional analysis investigated the association between dietary phenolic acids and cognitive status in older adults [12]. A randomized, double-blind, placebo-controlled, crossover clinical trial conducted on healthy middle-aged volunteers supplemented with fruit- and vegetable-based extract containing polyphenols reported significant improvements in executive function in terms of short-term memory, working memory, selective and sustained attention, and speed of processing when compared to the placebo group [10]. Similarly, another randomized, double-blind, placebo-controlled, crossover clinical trial conducted among healthy students reported that polyphenols-rich grape and blueberry extracts, implemented in the context of a healthy lifestyle, might be a safe alternative to acutely improved working memory and attention during a sustained cognitive effort [11]. Finally, a study conducted in a cohort of older Italian adults showed an association between the habitual dietary intake of phenolic acids (notably contained in coffee, berries, nuts, artichokes, and olive oil) and cognitive status, demonstrating that individuals with a lower intake of phenolic acids were more likely to have impaired cognition [12].

Several literature reviews aimed to comprehensively summarize the existing evidence on the effects of antioxidants, including polyphenols, toward cognitive and affective disorders, as well as mental health, and provide a mechanistic basis for their actions [13,14,15,16,17,18,19]. The findings confirmed the implications of diet and dietary antioxidants in the brain and mental health through various mechanisms, such as the modulation of neuroinflammation, adult neurogenesis, synaptic plasticity [13], as well as mitophagy [14]. Likewise, an overview was provided on the insights into how nutraceuticals may regulate cognitive function by targeting the TGF-β1 signaling pathway [15] and oxidative response [16]. In addition, an assessment of the preclinical and clinical studies on the effect of therapies able to reduce oxidative stress and mitochondrial alterations on the cognitive dysfunction associated with Down syndrome was published [17]. A review summarizing the effects of Royal jelly (RJ), the main food of queen bees, highlighted that it might promote brain cell survival and function by targeting multiple adversities in the neuronal microenvironment, such as inflammation, oxidative stress, mitochondrial alterations, and bioenergetic challenges [18]. Finally, a possible role of certain herbs, namely *Scutellaria baicalensis* (*S. baicalensis*), *Hericium erinaceus* (*H. erinaceus*), and *Rhodiola rosea* (*R. rosea*), in modulating neurotransmitter and neuroendocrine systems, stimulating neurogenesis and the synthesis of neurotrophic factors was emphasized [19].

The new insights on the effect of dietary antioxidants on brain disorders provided in this Special Issue are encouraging. Nonetheless, further research is needed to confirm and better understand the mental health benefits of dietary antioxidants.
